# Ginseng for improving semen quality parameters: A protocol of systematic review

**DOI:** 10.1097/MD.0000000000009732

**Published:** 2018-01-26

**Authors:** Hye Won Lee, Ki-Jung Kil, YoungJoo Lee, Myeong Soo Lee

**Affiliations:** a KM Convergence Research Division, Korea Institute of Oriental Medicine, Daejeon; b College of Oriental Medicine, Joongbu University, ChungNam; c Department of Bioscience and Biotechnology, College of Life Science, Sejong University, Seoul; d Clinical Research Division, Korea Institute of Oriental Medicine, Daejeon, Republic of Korea.

**Keywords:** ginseng, infertility, protocol, sperm, subfertility, systematic review

## Abstract

**Background:**

The aim of this systematic review is to determine whether ginseng is effective in improving sperm quality.

**Methods and analysis:**

Twelve databases will be searched from their inception to the present date: PubMed, EMBASE, AMED, the Cochrane Library, 5 Korean medical databases (KoreaMed, DBpia, OASIS, the Research Information Service System [RISS], and the Korean Studies Information Service System [KISS]), and 3 Chinese medical databases (China National Knowledge Infrastructure [CNKI], the Wanfang Database, and the Chinese Scientific Journals Database [VIP]). We will include all prospective clinical trials including randomized controlled trials (RCTs), controlled trials, and uncontrolled observational studies. We will exclude case study and case series, and retrospective studies in which healthy men or men with subfertility are treated with any type of ginseng. We will exclude studies comparing the 2 different forms of ginseng. The selection of the studies, data abstraction, and validations will be performed independently by 2 researchers. The risk of bias of the RCTs will be evaluated using the Cochrane's risk of bias assessment tool.

**Ethics and dissemination:**

The findings will be disseminated to appropriate audiences via peer-reviewed publications and conference presentations. Our review will provide readers the opportunity to access studies originally published in East Asian languages that they would otherwise be unable to read.

Trial registration number: PROSPERO 2017 CRD42017078797

## 1 Introduction

“Male factor infertility is defined as infertility caused primarily by male factors encompassing: abnormal semen parameters or function; anatomical, endocrine, genetic, functional or immunological abnormalities of the reproductive system; chronic illness; and sexual conditions incompatible with the ability to deposit semen in the vagina.”^[1]^ Many medical treatments have been introduced but their evidences of efficacy are unclear.^[2,3]^ The demands of using complementary and alternative medicine (CAM) are increasing as a solution for infertility including male factor infertility.^[4]^ Several studies reported the high usage of some form of CAM for improving fertility, libido, and sexual problems.^[5–8]^ One form of CAM is herbal medicine including danggui, maca, ginseng, etc.^[4,8–11]^

Ginseng is the root of the Panax plant can be administered in various forms, including tablets, liquid extracts, tinctures, powdered roots, sliced roots, and teas. This herbal medicine has many chemical constituents and bioactivities^[12]^ and has been used as a tonic to improve overall health, restore the body to balance, help the body heal itself, reduce stress, boost energy, and enhance the immune system.^[13]^ All of these effects are summarised by the term “adaptogen.”^[14]^ Adaptogens are agents that can promote resistance to external and internal stresses and improve both physical and mental faculties.

Several in vivo and in vitro studies have demonstrated that ginseng is effective for the spermatogenesis, improving testicular damage, sperm quality, and sperm mobility through cAMP-responsive element modulator.^[15–20]^ Ginsenosides are the active compounds responsible for the pharmacological effects of these plant extracts.^[21]^ Recent reviews reported that ginsenosides may affects the estrogen and androgen activities, and aphrodisiac activities.^[22]^ One systematic review showed suggestive effects of ginseng for erectile dysfunction possibly through the modulating humoral regulation or endothelial nitro oxide release. ^[23]^ However, the exact mechanisms of action of ginseng improving sperm parameter have not been clear yet.

However, to date, there has been no meta-analysis or systematic review on the use of ginseng for improving sperm quality. The objective of this systematic review is to determine whether ginseng is effective in improving sperm quality.

## 2 Methods

### 2.1 Study registration

This protocol review has been registered on PROSPERO 2017 CRD42017078797 (http://www.crd.york.ac.uk/PROSPERO/display_record.php?ID=CRD42017078797).

### 2.2 Criteria for considering studies for this review

#### 2.2.1 Types of studies

All prospective clinical trials including randomized clinical trial, controlled trials, and uncontrolled observational studies. We will exclude case study and case series, and retrospective studies.

#### 2.2.2 Types of participants

Healthy men or men with subfertility will be included regardless of age.

#### 2.2.3 Types of interventions

We will include any types of ginseng regardless of origin, age, processing status (e.g., fresh ginseng, white ginseng, or red ginseng) or dose. We will include trials comparing ginseng alone or co-intervention of conventional medicines with any types of controls including no-treatment, placebo, or conventional medicines.

#### 2.2.4 Types of outcome measures

Primary outcomes will be semen mobility, sperm concentration, and secondary outcomes will be sperm morphology, sperm count, semen volume, and adverse events.

### 2.3 Search methods for the identification of studies

#### 2.3.1 Electronic searches

Twelve databases will be searched from their inception to the present date: PubMed, EMBASE, AMED, the Cochrane Library, 5 Korean medical databases (KoreaMed, DBpia, OASIS, the Research Information Service System [RISS], and the Korean Studies Information Service System [KISS]), and 3 Chinese medical databases (China National Knowledge Infrastructure [CNKI], the Wanfang Database, and the Chinese Scientific Journals Database [VIP])

We will conduct a search of the relevant literature regardless of the language or publication status. For ongoing studies, we will search the following databases: the World Health Organisation (WHO), International Clinical Trials Registry Platform (ICTRP) (http://apps.who.int/trialsearch/), the Chinese Clinical Trial Registry (www.chictr.org), ISRCTN (www.controlledtrials.com/isrctn/), the National Institutes of Health Clinical Trials Database (www.clinicaltrials.gov), and the Clinical Research Information Service (CRiS) of the Republic of Korea. If necessary, we will contact the authors of the included studies and the researchers in the field to identify ongoing and unpublished studies.

#### 2.3.2 Other sources

We will also manually search relevant journals including such as *The Journal of Ginseng Research*. We will search for unpublished conference proceedings (e.g., Proceedings of the Ginseng Society Conference, and if possible, we will also review internal reports relevant to ginseng and the sperm quality. All included studies will be reviewed again to identify relevant bibliographic references. In addition, unpublished conference proceedings relevant to the sperm improvement will be reviewed if available.

#### 2.3.3 Search strategy

These strategies will be modified for use with other databases. The search terms used were “Panax ginseng OR ginseng,” AND “semen OR sperm OR hyposperm OR subfertility.” The search strategy was composed of a mixture of free text and thesaurus terms in Korean, Chinese, and English. The references in all located articles were manually searched for further relevant articles.

### 2.4 Selection of studies

Two review authors (HWL, KJK) will select inclusive articles by checking article titles and abstracts. These review authors will review hard copies of relevant publication to determine their inclusion. Any disagreements will be resolved through discussion and, if necessary, will use a third reviewer (MSL).

### 2.5 Data extraction and management

Two authors (HWL, KJK) will extract data from the selected reports or studies and independently complete a Summary of Findings table using the GRADEpro/GDT (https://gradepro.org/). We will extract information, such as the participants, interventions, outcomes and results, from each report. We will resolve disagreements by discussion and a third author (MSL) will act as an arbiter.

### 2.6 Assessment of risk of bias in included studies

Two authors (HWL and MSL) will independently assess the risk of bias in the included studies according to the guidelines in the Cochrane Handbook of Systematic Reviews of Interventions to evaluate the risk of bias (sequence generation, allocation concealment, blinding, incomplete outcome data, and selective outcome reporting).^[24]^

### 2.7 Data synthesis

We will present dichotomous data as a risk ratio (RR) with 95% confidence intervals (CIs). We will use the mean difference (MD) with 95% CIs for continuous data. We will use the standard mean difference (SMD) in cases of outcome variables with different scales. We will use the random-effects models for the meta-analysis. We will include principal data from the parallel group studies in the meta-analysis. We will use the data for analysis of the first session in the case of crossover studies. We will make an effort to contact the original authors of the study to obtain any missing or incomplete information if there are missing data needed for statistical analysis.

We will conduct a meta-analysis according to simultaneous use of the random-effects models if a significant number of studies are identified. We will include all studies in the primary analysis and conduct a sensitivity analysis by leaving out the studies with a high or unclear risk of bias. We will check for heterogeneity using the *I*^2^ statistic, which will indicate the number of inconsistencies among the included studies. We will use a 50% cut-off point for meaningful heterogeneity among the included studies. We will conduct subgroup analyses to identify the factors associated with heterogeneity (not predefined) if heterogeneity is observed. If we cannot perform a meta-analysis due to the clinical and methodological heterogeneity of the included studies, the findings of the review will be presented as a descriptive synthesis.

We will use funnel plots to detect reporting biases and small-study effects. We will conduct the test for funnel plot asymmetry using Egger's method if more than 10 studies are included in the meta-analysis.^[25]^

## 3 Discussion

Currently, no systematic review of ginseng for improving sperm quality exists. The fully completed systematic review will provide a summary of the current state of evidence regarding the effectiveness of the ginseng for sperm parameters. The review will be useful to patients and healthcare providers as well as patients.
